# The Effect of Pore Volume on the Behavior of Polyurethane-Foam-Based Pressure Sensors

**DOI:** 10.3390/polym14173652

**Published:** 2022-09-02

**Authors:** Mohammed Nabeel, Miklós Varga, László Kuzsella, Béla Fiser, László Vanyorek, Béla Viskolcz

**Affiliations:** 1 Institute of Chemistry, University of Miskolc, 3515 Miskolc-Egyetemváros, Hungary; 2 Ministry of Science and Technology—Materials Research Directorate, Baghdad 10011, Iraq; 3 Higher Education and Industrial Cooperation Centre, University of Miskolc, 3515 Miskolc-Egyetemváros, Hungary; 4 Institute of Materials Science and Technology, University of Miskolc, 3515 Miskolc-Egyetemváros, Hungary; 5 Ferenc Rakoczi II Transcarpathian Hungarian College of Higher Education, 90200 Beregszász, Transcarpathia, Ukraine

**Keywords:** pressure sensors, polyurethane (PU), nitrogen-doped, bamboo-shaped carbon nanotubes, N-BCNT, pore size, pressure sensitivity

## Abstract

In this work, three different polyurethane (PU) foams were prepared by mixing commonly used isocyanate and polyol with different isocyanate indices (1.0:0.8, 1.0:1.0, 1.0:1.1). Then, the prepared polyurethane foam samples were coated by dip-coating with a fixed ratio of nitrogen-doped, bamboo-shaped carbon nanotubes (N-BCNTs) to obtain pressure sensor systems. The effect of the isocyanate index on the initial resistance, pressure sensitivity, gauge factor (GF), and repeatability of the N-BCNT/PU pressure sensor systems was studied. The pore volume was crucial in finetuning the PU-foam-based sensors ability to detect large strain. Furthermore, large pore volume provides suitable spatial pores for elastic deformation. Sensors with large pore volume can detect pressure of less than 3 kPa, which could be related to their sensitivity in the high range. Moreover, by increasing the pore volume, the electrical percolation threshold can be achieved with a minimal addition of nanofillers. On the other hand, PU with a smaller pore volume is more suitable to detect pressure above 3 kPa. The developed sensors have been successfully applied in many applications, such as motion monitoring and vibration detection.

## 1. Introduction

Piezoresistive flexible pressure sensors (PFPS) that respond to mechanical stimulation received great attention in many applications such as motion detection [1,2], electronic skin [3,4], healthcare monitoring [5,6], and wearable electronic devices [7,8]. Traditional and commercially available metal- and silicone-based pressure sensors have good pressure sensitivity but these are only applicable either at high or low pressures, and inappropriate to use in flexible devices [9,10]. In addition, most sensors are expensive and not suitable for a long service life, due to their lack of flexibility [11]. Therefore, PFPSs produced by dip-coating are an effective sensor-type alternative, because they have a wide detection range, short response time [12], excellent durability, and low production cost [13,14].

For the design of PFPS with different features, it is important to select virgin materials with high flexibility. Various foam types have been used for the development of PFPS, such as PU [15,16,17], polydimethylsiloxane (PDMS) [18,19], and polyvinylidene fluoride (PVDF) [20]. Such types of foams can be used as substrates for nanofillers to obtain a highly flexible pressure sensor [21]. PU foam with high porosity [22] provides a suitable polymer matrix material for PFPS. In addition, PU has a modifiable macromolecular structure [23,24], and can be easily recycled and blended with other polymers [25,26,27], which makes it a suitable candidate for PFPS manufacturing [28,29] and other applications such as antibacterial water filter and acoustic insulation [30,31]. The development of polyurethane-foam-based PFPS involves dip-coating, during which the base material (PU) is immersed into a liquid phase containing the nanofiller. However, PFPSs prepared by dip-coating are usually unstable, due to the poor interaction between the nanofiller and PU. Several previous studies attempted to solve this issue. MXene–PU pressure sensors were developed with improved stability by coating the foam with positively charged chitosan and negatively charged Mxene, which increased the electrostatic interaction between the materials [21]. In our previous work, an N-BCNT–PU system was prepared and then impregnated with silicone rubber to improve the stability of the sensor [1]. Another approach is to change the properties of the PU, e.g., by using an excessive amount of isocyanate during the synthesis, which leads to a more rigid and more hydrophobic polyurethane [32]. On the other hand, PU samples prepared with higher polyol ratio are more hydrophilic, which could strengthen the interaction with the nanofiller (e.g., N-BCNTs) and enhance the stability of the system.

Conductive nanofillers in flexible polymers cause a change in electrical resistance in response to the applied pressure. The performance of these PFPSs depends on the electrical conductivity of the nanofiller. Therefore, nanofillers with excellent electrical conductivity, such as carbon nanotube (CNT) [33,34,35], graphene (GNP) [17,36], graphene oxide (GO) [37], carbon black [38,39,40], and their hybrids [41,42,43], have been extensively tested in PFPS preparation. Among different nanofillers, the CNTs are the most appropriate ones for pressure sensor design, due to their excellent electrical properties, and their unique fibrous structure [44]. 

Electrical resistance and pressure sensitivity have been extensively studied. Ma et al. fabricated a pressure sensor based on hybrid nanofillers (CNTs and GNP). It was found that the electrical resistance and pressure sensitivity improves compared to CNTs alone [11]. Additionally, the high number of dipping cycles during the preparation of a PU/GO system increases the sensitivity and conductivity of the sensor [45]. Another study investigates the effect of CNT loading on the PU/silicone rubber pressure sensor. It was found that the resistance of the sensor decreases when the CNT content increases, and, thus, the pressure sensitivity is improved [2]. In this study, a novel pressure sensor is developed that can be applied in a wide pressure range in both contact and non-contact modes. For the first time, the effect of the isocyanate index on the behavior of the PU-based pressure sensor was also investigated.

## 2. Materials and Methods

### 2.1. Materials

In the synthesis of N-BCNT, *n*-butylamine (C_4_H_11_N, Sigma Aldrich Ltd., D-14482 Hamburg, Germany), nickel nitrate hexahydrate (Ni(NO_3_)_2_*6 H_2_O, Thermo Fisher GmbH, D-76870 Kandel, Germany), magnesium oxide (MgO, Merck Ltd., D-64293 Darmstadt, Germany), and nitrogen (99.995% purity, Messer Ltd., H-1044 Budapest, Hungary) were used. PU was prepared by mixing isocyanate and polyol with different isocyanate indices (1.0:0.8, 1.0:1.0, 1.0:1.1). The reagents for PU foam production were Ongronat TR4040, which is a mixture of monomeric methylene diphenyl diisocyanate (MDI) and polymeric MDI (Wanhua-BorsodChem, H-3700 Kazincbarcika, Hungary), and Ongropur FFP-303 polyether-type polyol premix (Wanhua-BorsodChem, H-3700 Kazincbarcika, Hungary). The catalysts, blowing agents, surfactants, and other additives were included in the polyol component (Alcupol F2831, Repsol, 28045 Madrid, Spain). Patosolv (mixture of aliphatic alcohols, 98% ethanol, and 2% isopropanol, Molar Chemicals Ltd., H-2314 Halásztelek, Hungary) was used as a dispersant during the impregnation of polyurethane foams with N-BCNT.

### 2.2. CCVD Synthesis of Nitrogen-Doped, Bamboo-Shaped Carbon Nanotubes

The N-BCNT synthesis was carried out by using the catalytic chemical vapor deposition (CCVD) method with previously optimized synthetic parameters [46]. In the CCVD synthesis of N-BCNTs 5 wt.%, nickel-containing magnesium oxide catalyst was heated to 750 °C in a quartz tube, which was in a tube furnace. The synthesis lasted for 20 min and the carbon source (*n*-butylamine) was added at a rate of 6 mL h^−1^, while the nitrogen carrier gas flow was 100 mL min^−1^. The production cycle was repeated ten times. The N-BCNT sample was then purified, and the catalyst (magnesium oxide and nickel) was removed with concentrated hydrochloric acid (36 wt.%). The purity of the synthesized N-BCNT was evaluated by thermogravimetric analysis (TGA).

### 2.3. Preparation of PU Samples

**Figure 1:**
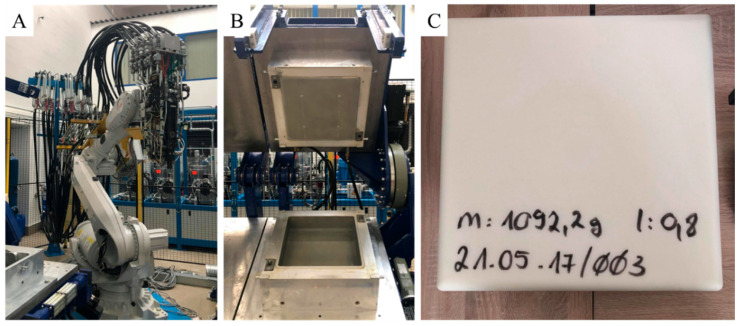
Robotic arm for the injection of polyurethane (PU) components (**A**), mold (**B**), and the prepared PU foam with 1.0:0.8 isocyanate index before post-processing and impregnation with N-BCNT suspension (**C**).

Three different types of PU foam samples were prepared by mixing isocyanate and polyol with different isocyanate indices. During the synthesis, the ratio of isocyanate groups to hydroxyl groups in the reaction mixture were 0.8:1.0, 1.0:1.0, and 1.1:1.0. The production of PU foams was carried out by the reaction of isocyanate and polyol, while water was also added as blowing agent to initiate the formation of CO_2_. Carbon dioxide transforms the polyurethane into PU foam. The isocyanate is the so-called hard segment, while the polyol is the soft segment in the PU. The structural, mechanical, and physical properties of the polymer are controlled by their ratio [47,48]. Increasing the hard segment (isocyanate) leads to an increase in resistance in PU [32]. It was found that sample 1, with a lower isocyanate index, has a higher pore volume content than sample 2 and sample 3. The PU samples were prepared using the Topline casting machine (Hennecke GmbH, Figure 1A–C). 

The machine operated with a high-pressure process and was suitable to produce flexible polyurethane foams. Mixing of the components (polyol and isocyanate) took place in the mixing head by a countercurrent injection process. The robotic arm with the high-pressure mixing head (Figure 1A) injected the reaction mixture of raw materials into the jacketed thermostatic aluminum mold (Figure 1B). The final size of the PU product is 400 × 400 × 100 mm. Then cylindrical samples (h = 32 mm, d = 29) were cut out and used to make the piezoresistive sensors.

### 2.4. Preparation of the N-BCNT/PU Nanocomposite

**Figure 2:**
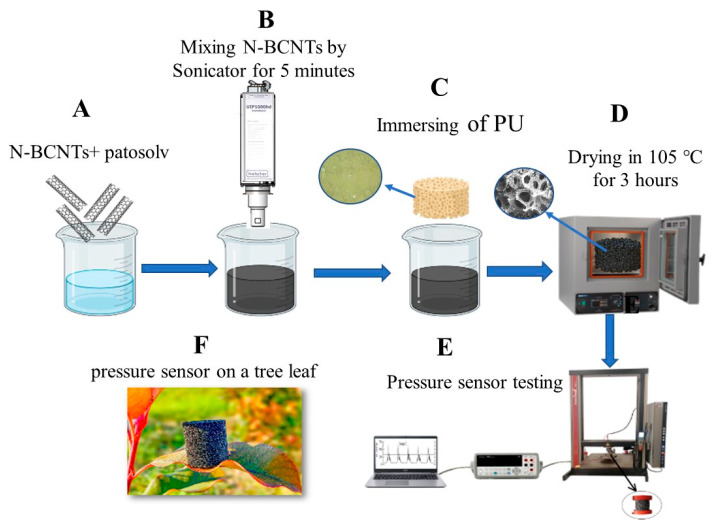
Schematic representation of the preparation and testing of the designed piezoresistive N-BCNT/PU sensor. (**A**)—mixing N-BCNT with alcohol, (**B**)—dispersion of N-BCNT in alcohol by applying sonication, (**C**)—immersion of PU samples into the N-BCNT dispersion, (**D**)—drying the N-BCNT/PU system, (**E**)—testing the pressure sensor, (**F**)—artistic picture of the pressure sensor.

The piezoresistive nanocomposite was prepared in several steps (Figure 2A–D). First, ≈0.147 g N-BCNT was dispersed in patosolv (100 mL) for 5 min using a Hielscher UIPHdt1000 tip homogenizer (340 W/19.42 kHz, Figure 2B). Then, the PU samples were immersed into the N-BCNT dispersions and after that, the samples were dried at 105 °C to evaporate patosolv and obtain the N-BCNT/PU pressure sensors (Figure 2C,D). Immersion and drying were repeated three times to maximize the amount of N-BCNT absorbed by the foam samples. The weight percentage of the added N-BCNTs to PU is the same for all samples (Table 1). The final N-BCNT/PU sensors differed in terms of their isocyanate index (Table 1).

### 2.5. Characterization Techniques

The synthesized carbon nanotubes were examined by high-resolution transmission electron microscopy (HRTEM) using a FEI Technai G2-20X Twin instrument (accelerating voltage: 200 kV). Sample preparation was performed by dropping the aqueous suspension of the samples onto a copper grid (300 mesh, carbon only from Ted Pella). The N-BCNT/PU foams were examined with a high-resolution scanning electron microscope (SEM). For this purpose, a Helios G4 PFIB Cxe (Thermo Fisher Scientific, Waltham, MA, USA) was used and the samples were prepared with carbon tape. During sample preparation, the foams were coated with gold sputtering. A Zeiss Discovery V12 stereo microscope was also used to examine the foam samples. After purification with acid, the carbon content (purity) of the N-BCNTs was determined by thermogravimetric analysis (TGA) using a Netzsch Tarsus TG 209 thermal microbalance. The combustion in pure oxygen was considered too fast, and, thus, a mixture of nitrogen and oxygen was used to burn the carbon content of the samples. Therefore, the TGA measurements were performed in a mixed atmosphere of nitrogen (4.5) and oxygen (5.0), with flow rates set to 6 mL/min and 14 mL/min for O_2_ and N_2_, respectively. The heating rate was 10 °C/min in the temperature range of 35 and 800 °C. The surface functional groups of N-BCNTs were identified by Fourier transform infrared spectroscopy (FTIR) using a Bruker Vertex 70 spectrometer. The prepared N-BCNT (2 mg) was added to 250 mg of spectroscopic potassium bromide and after homogenization, a pellet was prepared and used for transmission mode measurements. The Malvern Nano Zs instrument was used to measure the zeta potential (electrokinetic potential) of the N-BCNT, and the electrophoretic mobility was determined using the Smoluchowski equation. For this purpose, 2 mg N-BCNT was dispersed in 250 mL of distilled water in an ultrasonic bath. The chemical bonding configurations of the incorporated nitrogen atoms were determined by X-ray photoelectron spectroscopy (XPS) using a SPECS Phoibus 150 MCD nine analyzer. The N-BCNTs were examined by Raman microscopy (WITECH 3112973 instrument with HeNe laser, λ: 632.92 nm). On the Raman spectra of the nanotubes, two bands can be found, the defect peak (D-peak) around 1340 cm^−1^ and the graphite peak (G-peak) around 1580 cm^−1^. The ratio of these peaks’ intensities (I_D_/I_G_) are proportional to the structural defects

Micro-CT measurements were performed to study the pressure sensors by using a YXLON FF35 instrument (microfocus X-ray tube, transmission beam, accelerating voltage: 90 kV, Al phase: 0.5 mm, voxel size: 15.6 µm). The pores were analyzed with the porosity analysis/foam structure analysis modules of the VG Studio software after applying adaptive Gaussian filtering.

## 3. Results and Discussion

### 3.1. Characterization of the Synthesized N-BCNT

**Figure 3:**
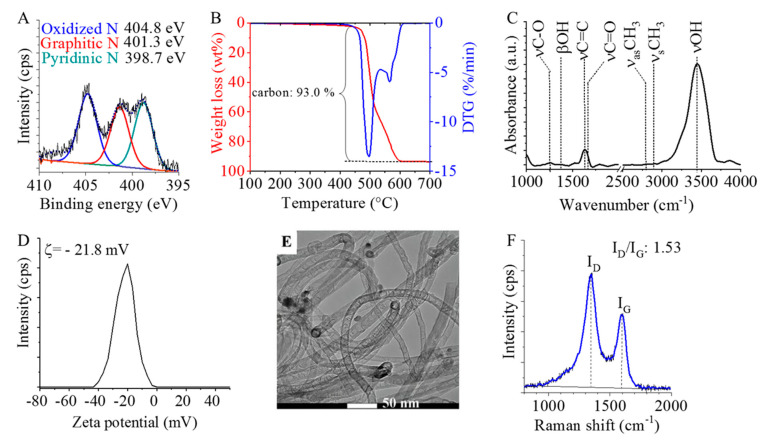
Characterization of the prepared N-BCNTs. XPS spectrum (**A**), TGA–DTG curves (**B**), FTIR spectrum (**C**), zeta potential distribution (**D**), TEM image (**E**), and Raman spectra with the D-peak and G-peak (**F**).

The incorporation of nitrogen into the structure of the nanotubes was confirmed by XPS measurements. Deconvolution of the N 1s band identified the C–N bond types in the N-BCNT structure (Figure 3A). In the XPS spectrum, three bands are found at 404.8 eV, 401.3 eV, and 398.7 eV, corresponding to the oxidized (pyridine N-oxide), graphitic, and pyridinic nitrogen, respectively. By changing the nitrogen content, the conductivity of the nanotubes can be altered [49,50]. TG analysis shows that the total carbon content (purity) of the prepared N-BCNT is 93 wt% (Figure 3B). Oxygen-containing functional groups (e.g., -OH and -COOH) are also present on the walls of the N-BCNTs according to infrared spectroscopy results (Figure 3C). These groups facilitate the dispersion of N-BCNTs into the liquid phase. At 1252 cm^−1^, a band is identified on the FTIR spectrum of the N-BCNT, and it can be associated with the C-O stretching vibrational mode of the hydroxyl and carboxyl groups. The βOH vibrational mode of the hydroxyl and carboxyl groups appear as an absorption peak around 1400 cm^−1^. The νC=O band is located at 1661 cm^−1^, which is interwoven with the C=C band and this peak indicates the presence of carbonyl or carboxyl groups. The skeletal vibration of the CNT structure and the νC=C stretching vibration are associated with a peak at 1631 cm^−1^ on the N-BCNT spectrum. Two more peaks are located at 2823 cm^−1^ and 2905 cm^−1^, and these belong to the asymmetric and symmetric stretching of the C–H bonds, respectively. A broad band is visible around 3400 cm^−1^, which can be associated with νOH. The zeta potential of the N-BCNT system is found to be −21.8 mV (Figure 3D). The hydroxyl groups can be deprotonated, leading to a decrease in the zeta potential and indicating good dispersibility, because the negatively charged surface stabilizes the aqueous N-BCNT dispersion. Moreover, the N-BCNTs disperse well in ethanol also (as polar solvent) and can form stable suspension. The stability of the ethanolic N-BCNT suspension ensures the homogeneous adsorption of nanotubes in the total volume of the PU matrix. Furthermore, the surface functional groups and surface polarity of N-BCNTs improve the sorption interaction with the PU matrix, which further supports the uniform distribution of the nanotubes on the surface of the foam. The fibrous structure of the synthesized N-BCNT can be seen in the HRTEM image of the purified sample (Figure 3E). The average diameter of the N-BCNTs outer tube is 19.7 nm. Catalyst-related impurities are not visible next to the nanotubes. The hemispherical fullerene-like building blocks of the N-BCNTs are also visible. Due to the incorporation of dopant nitrogen atoms, the nanotubes take on a characteristic bamboo-like structure. The nitrogen atoms and the bamboo-like structure induce several defects and vacancies in the graphitic structure of the N-BCNTs. Thus, the N-BCNTs are less graphitic, which means that the lattice structure is less ordered compared to conventional non-doped, multi-walled carbon nanotubes (MWCNTs). This disordered structure of the N-BCNTs was examined by Raman spectroscopy (Figure 3F). On the Raman spectra of the carbon nanotubes, two intensive peaks are found around 1340 cm^−1^ (D-peak) and 1580 cm^−1^ (G-peak), which correspond to the in-plane motion of the carbon atoms (Figure 3F) [51]. The D-peak indicates the presence of impurities or disorder in the carbon nanostructures, while the G-peak can be associated with the carbon-carbon bond stretching. The disordered structure of the nanotubes can be characterized by using the intensity ratio of these peaks. In case of our N-BCNT sample, the I_D_/I_G_ ratio is relatively high, 1.53. Due to the numerous lattice defects in their structure, these bamboo-like nanotubes are easily modifiable and, thus, various functional groups (e.g., -COOH, -OH) are located on their surfaces, which contributes to their high dispersibility in polar solvents and improves the adsorption interactions between the PU matrix and N-BCNTs. These properties are advantageous during the impregnation of the PU samples.

### 3.2. Electrical and Piezoresistive Properties of the Pressure Sensor

**Figure 4:**
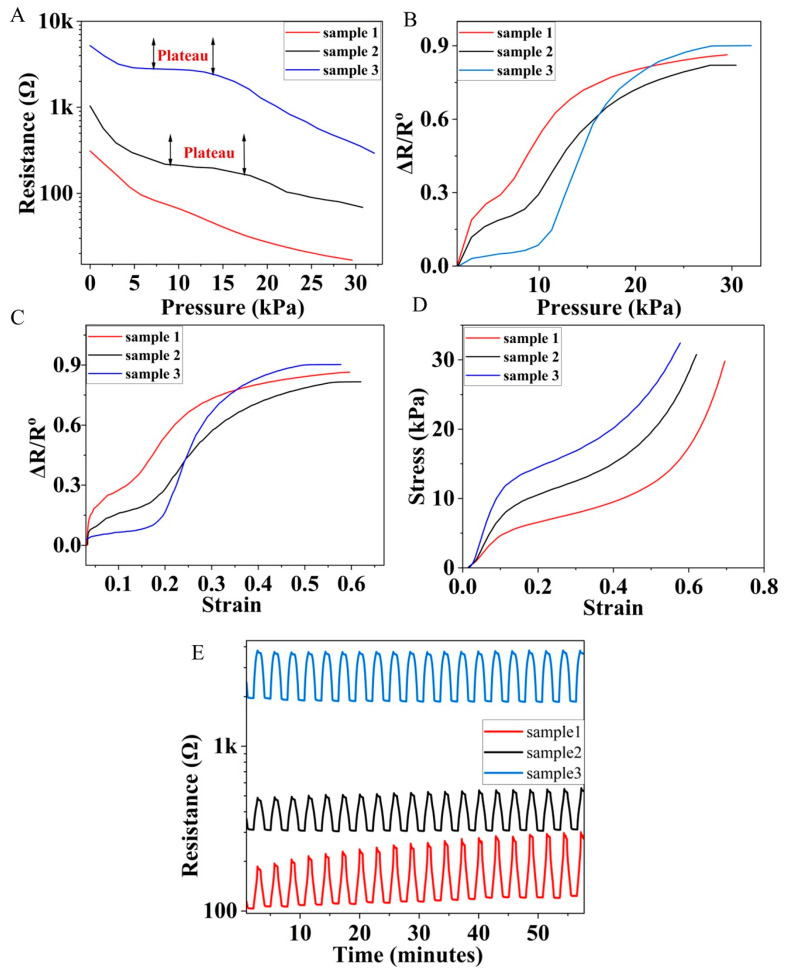
Resistance vs. pressure plots (**A**), pressure sensitivity (**B**), gauge factor (**C**), stress–strain curves (**D**), and cyclic load (**E**) of the developed N-BCNT/PU sensors (sample 1, sample 2, and sample 3).

The changes in electrical resistance of the N-BCNT/PU nanocomposites were studied in response to compression pressure (Figure 4A). The compression pressure was measured with a Zwick/Roell Z010-type universal electromechanical testing machine, and the electrical resistance was determined using an oscillator simultaneously. It was observed that the resistance decreases when the applied compression pressure increases. For example, in sample 1, the resistance drops from 108 Ω to 67 Ω when the pressure is increased from 5 kPa to 10 kPa. This could be related to the fact that the distance between the N-BCNTs becomes smaller, which leads to a more electrically conducive pathway in the PU system. In addition, the variation of the initial resistance of the samples is also recorded (Figure 4A). The initial resistance increases from 310 in the case of sample 1 to 1034 and 5200 in the case of sample 2 and sample 3, respectively.

**Figure 5:**
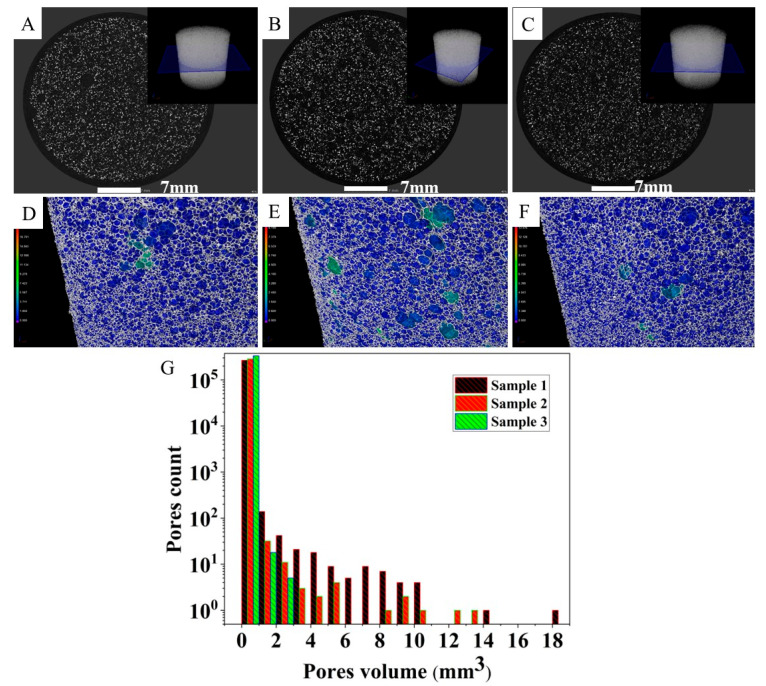
Micro-CT mapping plane and images of sample 1 (**A**,**D**), sample 2 (**B**,**E**), and sample 3 (**C**,**F**) along with the pore volume distribution (**G**).

This phenomenon indicates that the volume of the pores is a critical factor and could drastically change the resistance. Therefore, the pore volume of the samples (Figure 5), was investigated by using Micro-CT equipment to study the relationship between pore volume and resistance values. The total pore content of sample 1, sample 2, and sample 3 is 91.6, 85.3, and 78.1 vol. %, respectively. The most interesting result is that a sensor with larger pores has lower electrical resistance. This can be explained by the fact that the larger the pores, the smaller the overall PU scaffold and density. Hence, more N-BCNTs are interconnected, resulting in more conductive paths and a larger effective conductive area across the PU scaffold.

The plateau is described by a progressive compressive deformation at relatively constant compressive stress due to the elastic buckling of the PU scaffold [52,53]. The plateau region appears clearly in the ranges of 9–17 kPa and 7–14 kPa for sample 2 and sample 3, respectively (Figure 4A). On the other hand, there is no clear plateau in sample 1, which means that PU provides a suitable spatial for the elastic deformation of the foam due to the large pore volume.

The pressure sensitivity of the developed N-BCNT/PU piezoresistive sensors was measured (Figure 4B). The pressure sensitivity can be calculated according to following equation:

*S* = (Δ*R*/*R*_o_)/*P* (1)

where *S* is the sensitivity of the pressure sensor in kPa^−1^, Δ*R* = *R* − *R*_o_ is the resistance difference in Ω, *R*_o_ is the initial resistance in Ω, *P* is the applied pressure in kPa, and Δ*R*/*R*_o_ is the normalized resistance (NR). The *S* of sample 1 is higher than that of sample 2 and sample 3 in the 0–15 kPa range (Table 2). 

Sample 1 has a higher NR value than the other two samples. This could be due to the fact that sample 1 has a lower initial resistance. Therefore, high changes in the resistance in response to the applied pressure lead to a large variation in *S*. In contrast, sample 3 has a higher initial resistance than sample 1 and sample 2. Therefore, it is not very sensitive to small pressure changes (0–15 kPa). However, at higher pressures (15–30 kPa), sample 3 exhibits larger changes in pressure variation, yielding a larger NR value and, hence, a larger *S* value. The gauge factor (GF) of the developed N-BCNT/PU piezoresistive sensors was also determined (Figure 4C). GF can be calculated according to following equation:

GF = (Δ*R/R_o_*)/ε (2)

Δ*R* = *R* − *R*_o_ is the resistance difference in Ω, *R*_o_ is the initial resistance in Ω, *ε* is the strain, and Δ*R*/*R*_o_ is the normalized resistance (NR). 

GF in the strain range of 0.00–0.25 for sample 1 is higher than that of sample 2 and sample 3 (Table 2). As mentioned above, sample 1 has a high pore volume. Therefore, this phenomenon could be related to the fact that the increase in Δ*R*/*R*_o_ at a low compressive strain is mainly determined by the interconnection of the pores, which leads to the formation of a more effective conductive structure. In the strain range of 2.5–0.6, sample 3 has the highest GF compared to sample 2 and sample 1. This phenomenon could be related to the fact that at high compressive strain, the PU foam becomes denser, and the contact area increases. Hence, the sensor with the smaller pore volume (sample 3) plays a crucial role in reducing the Δ*R*/*R*_o_ of the foam structure.

The corresponding stress–strain curves were also recorded for the prepared sensors (Figure 4D). The compressive modulus of the sensors is 0.064, 0.095, and 0.15 MPa for sample 1, sample 2, and sample 3, respectively. Such results correspond to the high pore volume of sample 1, which make the stiffness decrease. Moreover, the compressive strength is also improved by increasing the isocyanate content. Thus, the compressive strength of the PU foam can be improved by increasing the isocyanate index. These results support the idea that decreasing the diisocyanate content leads to an increase in the total pores of the PU foam.

The repeatability and recoverability of the nanocomposite sensors were also investigated (Figure 4D). The pressure on the N-BCNT/PU nanocomposite sensors was repeatedly increased and removed at two minute intervals for 55 min with a ramp of 10% to 30%. The samples show different behavior in terms of output signal stability and peak amplitude. There are several fluctuations and a gradual increase in peak amplitude over time in sample 1 and sample 2 compared to sample 3. Sample 1 has a large pore volume and, thus, it has more N-BCNT layers deposited than sample 2 and sample 3. Hence, too many N-BCNT layers in the PU scaffold increases the probability that the system will fracture under cyclic pressure [54].

**Figure 6:**
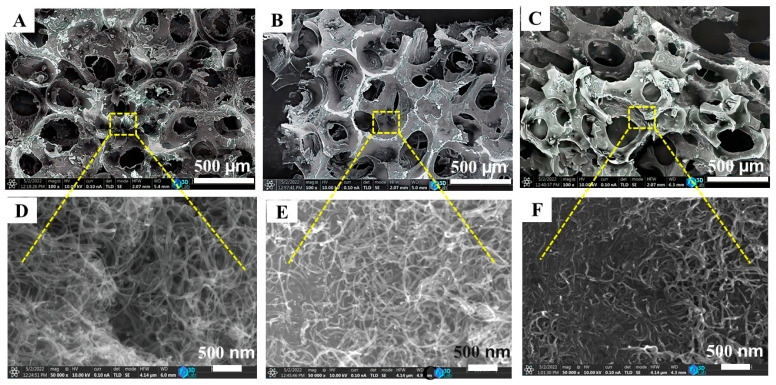
SEM images of the prepared N-BCNT/PU sensors, sample 1 (**A**,**D**), sample 2 (**B**,**E**), and sample 3 (**C**,**F**).

The morphology of the N-BCNT/PU was investigated (Figure 6), and it was found that the PU foams are completely coated with N-BCNT, and the structure reveals a wrinkle due to the presence of N-BCNTs. This could indicate that the nanotubes are bound to the foam by the dip-coating process. In addition, sample 1 is more wrinkled than samples 2 and 3, due to the larger total pore volume, which leads to a lower total PU scaffold and density. Consequently, more interconnected N-BCNTs lead to more wrinkles and burrs. The burrs and wrinkles serve as “microswitches”, which regulate the electrical resistance [55]. Therefore, the electrical resistance of sample 1 is expected to be lower than that of sample 2 and sample 3. In addition, the surface of sample 1 is rich and covered with more carbon nanotubes. Thus, sample 1 has a higher electrical conductivity and lower electrical resistance than sample 2 and sample 3. 

**Figure 7:**
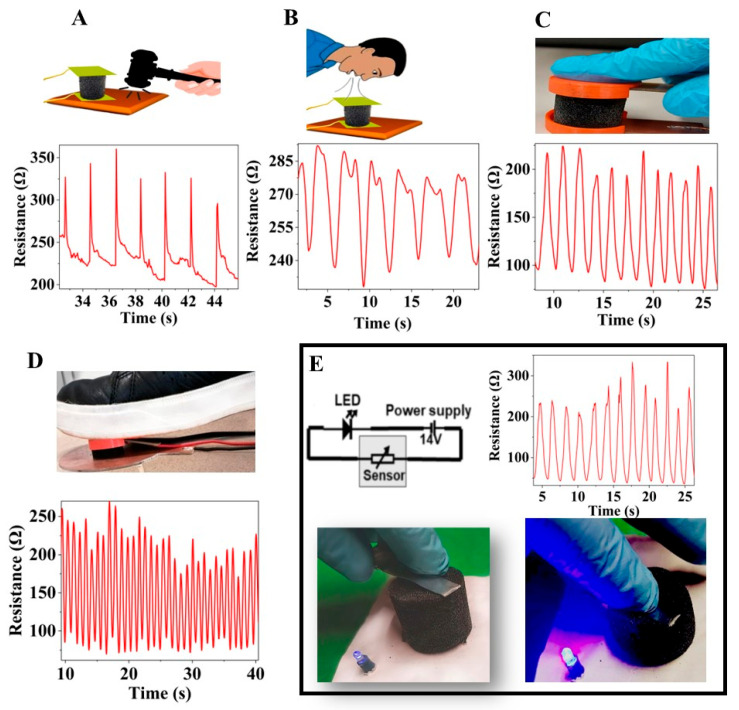
The developed N-BCNT/PU (sample 1) pressure sensor were tested in vibration detection (**A**), breath detection (**B**), finger touch detection (**C**), motion detection (**D**), and a LED-containing pressure sensor circuit was successfully created (**E**).

The PU/N-BCNT pressure sensors were tested in different applications in order to prove their applicability. The developed sensor (sample 1) records different level of pressure for different human activity, such as motion detection, finger touch detection, vibration detection, and breath detection (Figure 7). The peak amplitude of the change in resistance represents the vibration caused by the light tapping of the hammer (Figure 7A). The sensor shows high performance in detecting the low-pressure range, even for non-contact pressure modes such as the detection of breath (Figure 7B). Moreover, gentle breathing results in a nearly identical and repetitive change in resistance, while rapid breath leads to irregular patterns. In addition, the detection of motion and finger touch are also possible (Figure 7C,D). The resistance changes in the sensor were recorded during the pressure sequence during loading and unloading. The amount of pressure applied to the sensor during each movement with the finger or foot determines the peak amplitudes. This demonstrates the developed sensors’ ability to detect different types of motion. To prove the piezoresistive effect of one of the pressure sensors, a LE-containing pressure sensor circuit was also created by using sample 1 (Figure 7E). The brightness of the light from the LED increases when pressure is applied to the sensor. This also shows that the developed N-BCNT/PU nanocomposite has a piezoresistive effect and can be utilized as a pressure sensor. All in all, the properties of the developed sensors are very promising, and indicate that these could be utilized in wearable device applications.

## 4. Conclusions

Flexible pressure sensors were successfully developed, using PU foam as a support material for N-BCNTs. Three polyurethane foams with different isocyanate indices (0.8, 1.0, and 1.1) were successfully synthesized and then were used as skeletons and immersed into N-BCNT dispersion to create the N-BCNT/PU composite piezoresistive sensors. The initial resistance of the three different samples is 310, 1043, and 5200 Ω. Larger total pore volume leads to a lower initial electrical resistance, because the density of the PU scaffold is smaller. Consequently, more N-BCNTs are interconnected, resulting in more conductive paths and a larger effective conductive area in the PU scaffold. The increase in N-BCNT layers in the PU scaffold also increases the probability of fracture under cyclic compressive loading. As the pore volume of the pressure sensor increases, the pressure sensitivity of the sensor initially increases and then decreases as a function of the pressure. The sample with larger pore volume shows higher sensitivity at low compressive strain, but lower sensitivity at high compressive pressure. Monitoring of both small and large human activities by the developed pressure sensors is possible, which means that the N-BCNT/PU pressure sensors have high sensitivity and reliability.

## Figures and Tables

**Table 1:** N-BCNT and PU weight of the prepared N-BCNT/PU nanocomposite.

	Polyol: Isocyanate Ratio	N-BCNT Weight	PU Weight	N-BCNT wt.%
Sample 1	1.0:0.8	0.146 g	1.46 g	10%
Sample 2	1.0:1.0	0.147 g	1.47 g	10%
Sample 3	1.0:1.1	0.141 g	1.41 g	10%

**Table 2:** Sensitivity (S) and gauge factor (GF) of all samples in different pressure ranges.

Samples	S in Pressure Range 0–3 kPa^−1^	S in Pressure Range 6–15 kPa^−1^	S in Pressure Range 16–30 kPa^−1^	GF in Strain Range (0.00–0.08)	GF in Strain Range (0.18–0.27)	GF in Strain Range (0.27–0.57)
Sample 1	0.12 kPa^−1^	0.05 kPa^−1^	0.008 kPa^−1^	3.17	2.87	0.58
Sample 2	0.08 kPa^−1^	0.04 kPa^−1^	0.017 kPa^−1^	1.68	3.91	1.09
Sample 3	0.02 kPa^−1^	0.09 kPa^−1^	0.019 kPa^−1^	0.75	4.58	1.27

## Data Availability

The data presented in this study are available on request from the corresponding author.
